# Low-cost biomimetic sensor based on copper porphyrin-modified graphite electrodes for electrochemical detection of glyphosate in aqueous samples

**DOI:** 10.1039/d5ra05306d

**Published:** 2025-08-26

**Authors:** Ana M. Janeiro Tudanca, Rolando M. Caraballo, Facundo C. Herrera, Paula Giudici, Mariana Hamer

**Affiliations:** a Instituto de Ciencias, Universidad Nacional de General Sarmiento Los Polvorines Argentina; b INEDES, UNLu-CONICET Luján Argentina; c Laboratorio Argentino Haces de Neutrones-CNEA Villa Maipú Argentina; d Departamento de Física de la Materia Condensada, Instituto de Nanociencia y Nanotecnología, CNEA-CONICET, Centro Atómico Constituyentes San Martín Argentina; e Instituto de Ciencias, Universidad Nacional de General Sarmiento - CONICET Los Polvorines Argentina mhamer@campus.ungs.edu.ar

## Abstract

We present a biomimetic electrochemical sensor for glyphosate (GLY) detection, utilizing graphite electrodes modified with electropolymerized copper(ii) meso-tetra(4-sulfonatophenyl)porphyrin (CuP). The Cu(ii) centers provide dual functionality: catalytic oxygen reduction and selective GLY coordination, which leads to a proportional suppression of redox currents. Characterization (SEM-EDS/Raman/UV-Vis) confirmed CuP polymerization and specific GLY binding. The sensor achieved a 1 μmol L^−1^ detection limit (S/N = 3) with linear response (2–120 μmol L^−1^; RSD = 0.7%) and >98% recovery in spiked rainwater. Stability tests showed 99% signal retention after 30 days, outperforming enzyme-based sensors. This platform combines three key advantages: (1) sustainable fabrication ($0.12/electrode), (2) rapid analysis (<5 min per sample), and (3) field-deployability without instrumentation. The nanostructured CuP film improves sensitivity and contributes to selective GLY detection by excluding common interferents (nitrate/humic acid). Compared to chromatographic methods, this approach offers an eco-friendly alternative for environmental GLY monitoring.

## Abstract 2

We present a biomimetic electrochemical sensor for glyphosate (GLY) detection, utilizing graphite electrodes modified with electropolymerized copper(ii) meso-tetra(4-sulfonatophenyl)porphyrin (CuP).

## Graphical abstract

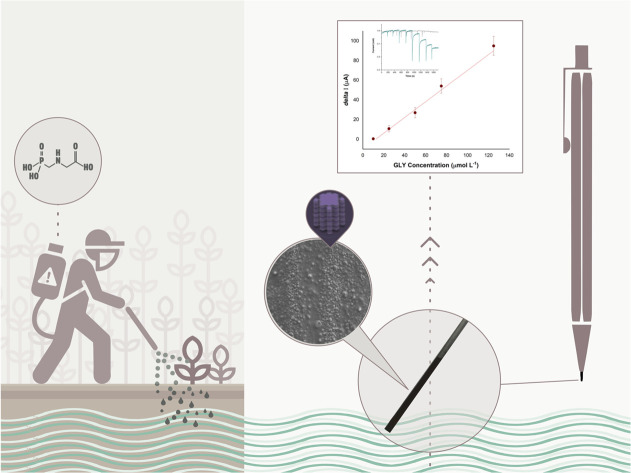

## 1. Introduction

Glyphosate (*N*-phosphonomethylglycine, GLY) is the world's most widely used organophosphate herbicide, with global agricultural reliance exceeding 800 000 metric tons annually.^1^ Its effectiveness in broadleaf weed control has resulted in widespread environmental contamination, with residues detected in 80% of European waterways,^2^ linked to ecological disruption,^3^ and associated with potential human health risks.^4^ Despite increasing regulatory scrutiny, conventional GLY detection methods, such as chromatography,^5^ ELISA,^6^ and fluorescence assays,^7^ remain constrained by high costs, laboratory dependence, and complex sample preparation. These limitations highlight the urgent need for portable, low-cost sensors capable of on-site monitoring to inform timely environmental and public health interventions.

Electrochemical sensors offer a compelling alternative, combining affordability, miniaturization, and rapid response.^8–10^ However, GLY's non-electroactive nature necessitates indirect detection strategies, often leveraging its strong affinity for metal ions like Cu(ii).^11,12^ Recent advances exploit Cu-based materials (*e.g.*, MOFs,^13^ oxides^14^) to mediate GLY sensing, but these systems face challenges in cost, stability, or fabrication complexity. Biomimetic approaches, inspired by natural metalloenzymes, present an untapped opportunity to address these limitations. Porphyrins—nature's quintessential redox-active macrocycles—exhibit tunable metal-binding sites and catalytic versatility, mirroring peroxidase enzymes.^15,16^ Notably, Cu(ii)-porphyrins mimic the active centers of laccases and catechol oxidases,^17,18^ enabling oxidative catalysis while selectively coordinating GLY *via* its amine, carboxylate, and phosphate groups.^19^ Prior work has harnessed porphyrin-MOF hybrids for GLY detection,^20,21^ yet these designs rely on expensive substrates (*e.g.*, gold nanoparticles) or lack scalability.

Here, we report a low-cost, biomimetic electrochemical sensor based on electropolymerized copper porphyrin (CuP) films on disposable graphite pencil electrodes (GPEs). Our design leverages CuP's dual function: (1) peroxidase–mimetic activity for catalytic signal amplification and (2) high-affinity Cu(ii)-GLY coordination to suppress redox signals proportionally to GLY concentration. Unlike existing sensors, this system eliminates costly nanomaterials while achieving a detection limit of 1 μM, 30 days stability, and 99% accuracy in spiked rainwater. By integrating biomimicry with scalable electrode fabrication, we bridge the gap between laboratory-grade sensitivity and field-deployable practicality. This work not only advances GLY monitoring but also establishes a template for metalloporphyrin-based sensing of other metal-coordinating pollutants.

## 2. Materials and methods

### 2.1 Chemicals and reagents

All chemicals were of analytical grade and used without further purification. Copper(ii) 5, 10, 15, 20-[meso-tetra(*N*-sulfate-phenyl) porphyrin] (CuTPPS) was purchased from Frontier Scientific. GLY (≥96%), sodium hydroxide (NaOH, 99%), 3,3′,5,5′-tetramethylbenzidine (TMB), hydrogen peroxide (H_2_O_2_, 30%), and potassium nitrate (KNO_3_) were obtained from Merck. Phosphate buffer saline (PBS, 250 mM, pH 7.2) was prepared using Na_2_HPO_4_ and NaH_2_PO_4_ (Sigma-Aldrich). Graphite pencil electrodes (GPEs, HB 0.7 mm) were sourced from Staedtler. Ultrapure water (18.2 MΩ cm) from a Millipore system was used for all solutions.

### 2.2 Instrumentation

The Scanning Electron Microscopy (SEM) images of the CuP polymer were taken with a Zeiss LEO1450VP. The energy dispersive analysis of the X-ray spectroscopy (EDS) studies was performed on an EDAX Genesis 2000. The samples were mounted on gold-coated carbon adhesive tape.

Raman scattering measurements were performed in a backscattering geometry at room temperature using as excitation source the 514.5 nm line of an Ar^+^ laser, and the spectra analysed with a Horiba Jobin Yvon LabRAM HR-800 spectrometer with a 1800 g per mm grating. The laser is focused on the sample, as well as the scattered light is collected using a microscope objective (WD = 10.6 mm, NA = 0.25) with 100 fold magnification, resulting in a laser spot size of 1 μm.

An HP8452 diode array spectrophotometer and a quartz crystal cell were used to obtain the UV spectra of CuP. A 1 cm^2^ geometric area of CuP functionalized ITO working electrode was measured on a holder designed for this purpose.

Cyclic voltammetry (CV), amperometry, and electrochemical impedance spectroscopy (EIS) were performed using a TEQ4-Z potentiostat (TEQ-Argentina) with a three-electrode system (WE: E or E/CuP, RE: Ag/AgCl (KCl 3 M), CE: Pt foil). All the electrochemical experiments were performed in the presence of oxygen.

### 2.3 Electrode fabrication

A 0.25 mg per mL CuTPPS solution in 0.1 M NaOH was prepared. 15 CV scans (0.0 to +1.4 V *vs.* Ag/AgCl, 50 mV s^−1^) were applied to deposit CuP onto GPEs.^22^ Electrodes were rinsed with ultrapure water and air-dried at room temperature for 5 min. The electrodes were stored at room temperature in the dark to preserve the stability and integrity of the porphyrin layer after modification, since porphyrins are known to be photosensitive compounds that can undergo photodegradation or photochemical reactions upon light exposure.

### 2.4 Electrochemical characterization

The active surface area was calculated *via* Randles–Sevcik equation using 25 mM K_3_[Fe(CN)_6_] in 0.1 M KNO_3_ (scan rates: 10–200 mV s^−1^). And, EIS analysis was conducted in 5 mM K_3_[Fe(CN)_6_]/K_4_[Fe(CN)_6_] (0.1 M KNO_3_) at +0.25 V (*vs.* Ag/AgCl), 100 kHz–0.1 Hz, 10 mV amplitude. Data fitted to a Randles circuit (ZView software).

### 2.5 Spectroscopic validation of GLY binding

CuP films deposited onto ITO were exposed to GLY (0–100 μM) in PBS (pH 7.2) and UV-Vis spectra were recorded to monitor Soret (400 nm) and Q-band (556 nm) shifts. E/CuP electrodes were also analyzed by Raman before/after GLY exposure (514 nm laser). Peak assignments followed literature for porphyrin vibrational modes.^17,18^

### 2.6 Biomimetic peroxidase activity assay

The interaction of GLY with the CuP was studied by following the colorimetric oxidation reaction of 3,3,5,5-tetramethylbenzidine (TMB) and H_2_O_2_.^23^ 2 μL TMB 40 mM, 2 μL H_2_O_2_ 0.89 M, and different concentrations of GLY were added to 0.1 M acetate buffer pH 5 until reaching a final volume of 200 μL. Modified and bare electrodes were immersed in these solutions and left in contact for 10 min. Then the absorbance at 650 nm (TMB oxidation product) was measured. A blank was performed adding ultra-pure water instead of GLY.

### 2.7 Amperometric GLY detection

Amperometric titration was done in a conventional three-electrode setup with the working potential polarized at 0.065 V Vs Ag/AgCl in 250 mM of phosphate buffer pH 7.2.^24,25^ This pH was selected because glyphosate is fully deprotonated and forms a 1 : 1 complex with Cu^2+^, which is the only species present in solution.^19^ The solution was gently stirred with a magnetic bar during the assays performed after successive additions of GLY (1 mM). Calibration was performed by plotting the net current response (Δ*I* = *I*_0_ − *I*) *versus* GLY concentration, where *I*_0_ is the baseline current in buffer and *I* is the current recorded after each addition of GLY.

Selectivity was tested against common potential interferents: nitrate, calcium ions, and humic acid.

### 2.8 Real-sample analysis

Spiked rainwater samples were diluted 1 : 1 with PBS and analyzed without further pretreatment. Samples were spiked with GLY (1.5–45 μM) and measured in triplicate (*n* = 3). Accuracy was calculated as (found/added) × 100.

## 3. Results and discussion

### 3.1 Characterization of CuP-modified electrodes

**Fig. 1:**
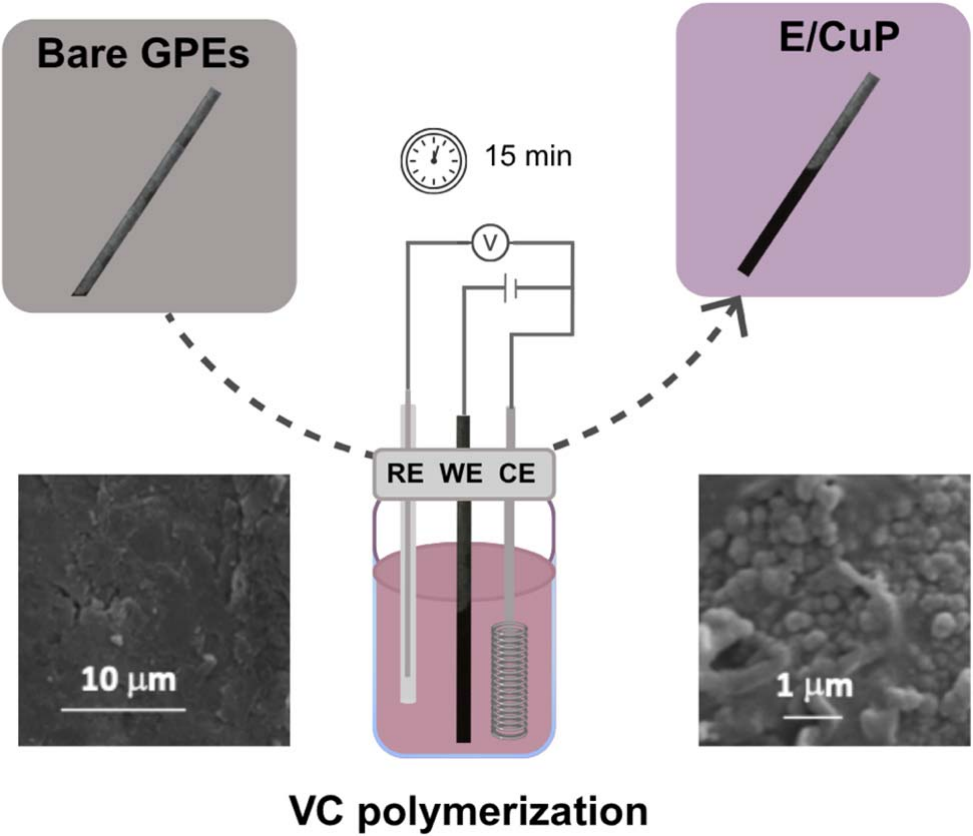
Diagram of the graphite electrode modification process by CuP electropolymerization, showing key steps from preparation to characterization.

The working electrodes were modified with a CuP (Fig. S1) polymeric film using a CV range between 0 and 1400 mV (Fig. S2). Fig. 1 depicts the simple and rapid modification process of the GPEs.

The morphological characteristics of the deposited film were examined using scanning electron microscopy (SEM) (Fig. S3). SEM images revealed that electropolymerized CuP formed an amorphous, microglobular film on graphite electrodes (E/CuP), uniformly covering the surface (Fig. S3B and C). In contrast, bare graphite (E) (Fig. S3A) showed a flat, layered structure. Additionally, elemental analysis of the modified electrodes was performed using energy-dispersive X-ray spectroscopy (EDS), providing detailed insights into the composition of the deposited material. EDS analysis (Table S1) confirmed the presence of Cu (0.8% wt) and S (5.45% wt), validating successful CuP deposition. The observed P and Cl signals (1.3% and 0.39% wt, respectively) likely originated from residual electrolyte or porphyrin sulfonate groups.

The electron transfer properties of bare and CuP-modified electrodes were evaluated using CV in 0.1 M KNO_3_ containing 25 mM Fe(CN)_6_^3−^/Fe(CN)_6_^4−^ as a redox probe. As shown in Fig. S4, all electrodes exhibited quasi-reversible voltammetric behavior, with anodic and cathodic peak currents (*I*_p_) demonstrating linear dependence on the square root of scan rate (*v*^1/2^) (*R*^2^ > 0.99). This relationship confirms a diffusion-controlled process, in agreement with the Randles–Sevcik equation (eqn (1)):^26^

1 *I*_p_ = 2.69 × 10^5^*n*^3/2^*A C*_i_* *D*_i_^1/2^*v*^1/2^

where *n* = 1 (electron transfer number), *A* is the electroactive area (cm^2^), C* is the bulk concentration (mol cm^−3^), and *D* = 6.70 × 10^−6^ cm^2^ s^−1^ (diffusion coefficient for Fe(CN)_6_^3−^).

Comparative analysis revealed significant differences between electrode configurations. A batch of three different E/CuP electrodes showed similar values (0.082 ± 0.001 cm^2^), whereas an active area of 0.19 ± 0.05 cm^2^ was calculated for the bare graphite electrode, showing a 37% reduction in active surface area. This decrease in active surface area indicates that the electropolymerized CuP film forms a uniform, semi-conductive coating that partially blocks access to the graphite substrate.

Additionally, EIS were employed to characterize the interfacial properties of both E and E/CuP electrodes in 5 mM Fe(CN)_6_^3−^/Fe(CN)_6_^4−^ solution.^27^ The Nyquist and Bode plots (Fig. S5) revealed fundamental differences in charge transfer mechanisms between the two systems.

The electrochemical behavior of the electrodes was modeled using a simplified Randles equivalent circuit (Fig. S5A, inset). Two main kinetic processes were identified. The first is the charge transport within the polymer film, represented by the film resistance (*R*_u_). The second is the heterogeneous electron transfer to Fe(CN)_6_^3−^, governed by the charge transfer resistance (*R*_ct_), the double-layer capacitance (*C*_dl_), and the diffusional impedance (*Z*_w_, Warburg element). The values of the circuit elements for each electrode are summarized in Table 1. Both bare and modified graphite electrodes conform to the same equivalent circuit but with variations in the contributions of individual components.

**Table 1:** Values of the Randles equivalent circuit elements for each electrode in Fig. S5^a^

*n*:1	*R*_u_ (kΩ)	*R*_ct_ (kΩ)	*C*_dl_ (μF)	*Z*_w_ (kσ)
E	13.5	225.8	1.1	277.1
E + GLY	13.7	271.2	7.6	232.5
E/CuP	15.6	803	9.7	1104
E/CuP + GLY	11.9	613.2	8	1121.3

a *R*_u_ = electrolyte resistance; *R*_ct_ = charge transfer resistance; *C*_dl_ = double-layer; *Z*_w_ = Warburg impedance.

Unmodified graphite electrodes exhibited typical fast electron transfer kinetics, as evidenced by their small semicircular region in the Nyquist plot (*R*_ct_ = 225.8 kΩ) and comparable Warburg diffusion impedance (*Z*_w_ = 277.1 kΩ). This behavior was further confirmed by Bode phase angle plots, which showed a pronounced 70° shift at high frequencies, characteristic of strong capacitive double-layer formation.

In contrast, CuP-modified electrodes demonstrated significantly altered impedance behavior. The modification resulted in a 3.6 – fold increase in charge transfer resistance (*R*_ct_ = 803 kΩ) and a 4-fold enhancement of Warburg impedance (*Z*_w_ = 1104 kΩ), clearly demonstrating that the electropolymerized CuP film acts as a semi-conductive barrier to electron transfer. The smoother phase transitions observed in Bode plots for modified electrodes, along with the increased double-layer capacitance (from 1.1 μF to 9.7 μF), suggest a transition from capacitive-dominated to resistive-dominated behavior, with charge transport occurring primarily through tunneling between Cu(ii) centers in the porphyrin matrix.^28,29^

Interestingly, the effects of GLY exposure on electrode impedance provided critical insights into the sensing mechanism. For CuP-modified electrodes, GLY complexation reduced *R*_ct_ by 24% (to 613.2 kΩ), which can be attributed to two complementary effects: neutralization of positive charges on the CuP surface that normally attract the anionic Fe(CN)_6_^3−^ probe, and enhanced electron tunneling through the newly formed Cu-GLY coordination spheres.^30^ Conversely, bare graphite electrodes showed a 20% increase in *R*_ct_ upon GLY exposure, likely due to non-specific adsorption of GLY molecules. These results highlight the crucial role of Cu(ii)-GLY coordination in the sensor's charge transfer modulation.

### 3.2 Spectroscopic validation of GLY binding

The interaction between GLY and the CuP film was investigated through complementary UV-Vis and Raman spectroscopy techniques. For UV-Vis studies, CuP was electropolymerized onto transparent ITO substrates to enable direct optical characterization. The absorption spectrum of the CuP film (Fig. S6) displayed characteristic porphyrin features: a sharp Soret band at 401 nm and distinct Q-bands at 556 nm, confirming successful film formation. Upon exposure to GLY, the Soret band underwent a 5 nm red shift (to 406 nm), while the Q-band similarly shifted. This red shift suggests strong coordination of GLY to the Cu(ii) center, likely through the phosphonate group, which alters the porphyrin's electronic structure. Notably, solution-phase CuP showed no spectral changes upon GLY addition (Fig. S7), demonstrating that binding requires the organized polymeric structure of the surface-immobilized porphyrin.

**Fig. 2:**
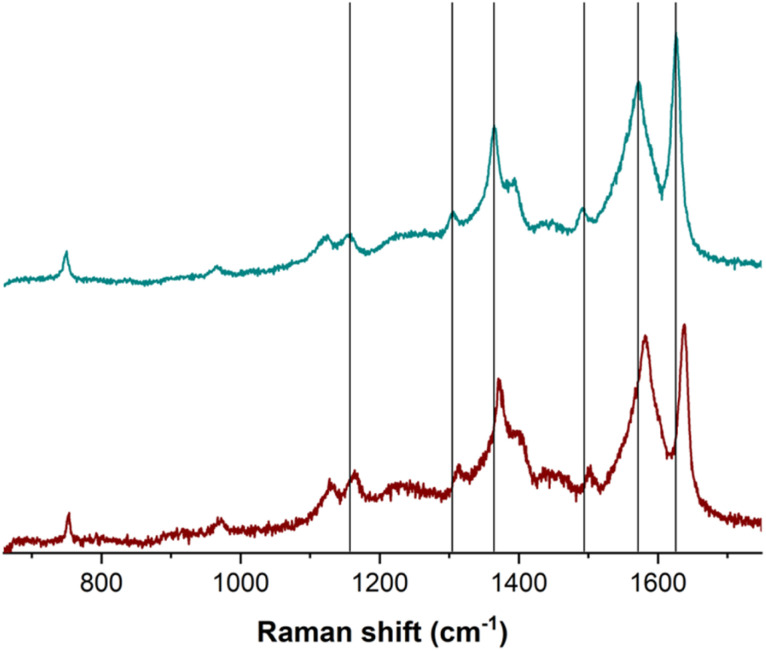
Raman spectrum of E/CuP (wine) and E/CuP + GLY (green). *λ*_exc_ = 514 nm.

Raman spectroscopy provided further molecular-level insights into the GLY–CuP interaction. The spectrum of unmodified E/CuP electrodes (Fig. 2 and S8) showed key vibrational modes of the porphyrin macrocycle, including: pyrrole breathing modes (750 cm^−1^), Cα-N symmetric stretching (1370 cm^−1^) and C=C stretching vibrations (1501, 1581, and 1638 cm^−1^). After GLY exposure, these characteristic peaks exhibited consistent 10–12 10–12 cm^−1^ downshifts (Table S3) most notably for the C=C stretching modes (1638 → 1626 cm^−1^). This uniform shift pattern reflects distortion of the porphyrin ring due to GLY coordination with the central Cu(ii) ion, altering the vibrational potential of the conjugated system. The magnitude of these shifts aligns with prior reports of axial ligand binding to metalloporphyrins,^31,32^ providing strong evidence for the formation of a Cu(ii)-GLY complex at the electrode surface.

The combination of these spectroscopic techniques conclusively demonstrates that GLY binds selectively to the Cu(ii) centers in the polymeric film, with both methods showing changes consistent with metal–ligand coordination rather than non-specific adsorption. This surface-mediated interaction forms the basis for the sensor's selectivity, as the organized porphyrin matrix preferentially coordinates GLY's unique combination of amine, carboxylate, and phosphonate functional groups.

### 3.3 Biomimetic activity assessment

**Fig. 3:**
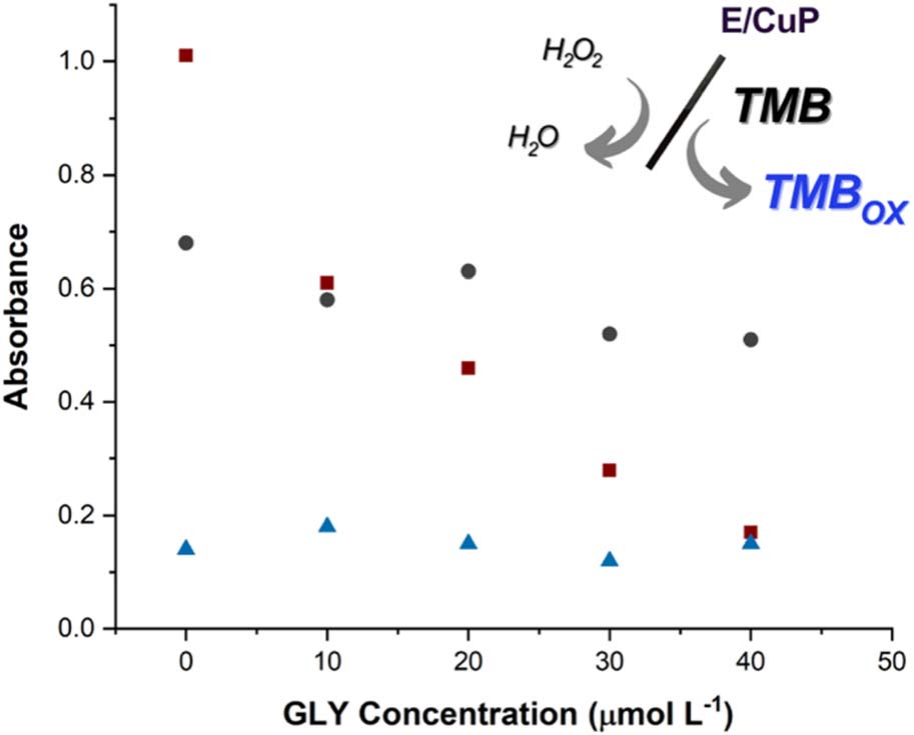
GLY concentration effect on the TMB oxidation assay. *A*_650_*vs.* GLY concentration measured 15 minutes after the reaction begins (0, 10, 20, 30, 40 μmol L^−1^). Red square: E/CuP; black circle: E; blue triangle: without electrode. Photos of the solutions in each point are shown in Fig. S9.

The CuP-modified electrodes exhibit intrinsic peroxidase-like activity, mimicking the function of natural heme-containing enzymes through their copper-porphyrin centers.^33^ This biomimetic property enables catalytic oxidation of the chromogenic substrate TMB in the presence of H_2_O_2_, producing the characteristic blue-colored TMB_ox_ with distinct absorption maxima at 370 nm and 650 nm (Fig. 3 inset).^34^

The inhibition assay revealed a dose-dependent response to GLY (0–40 μM), as observed in Fig. 3, with three key observations. First, unmodified graphite electrodes (E) showed minimal TMB_ox_ production, confirming the essential role of CuP in catalysis. Second, CuP-modified electrodes (E/CuP) generated significant TMB_ox_ (*A*_650_ ≈ 0.8), demonstrating effective peroxidase–mimetic activity. And, third, GLY addition caused up to 80% suppression of TMBox formation, with complete inhibition at 40 μM GLY, demonstrating competitive inhibition at Cu(ii) sites.

This competitive inhibition occurs through specific coordination of GLY to the Cu(ii) active sites, which normally catalyze H_2_O_2_ reduction to hydroxyl radicals. The phosphonate group of GLY preferentially binds to Cu(ii), blocking the enzyme-like pocket and preventing the redox cycling essential for TMB oxidation. The linear correlation between GLY concentration and signal suppression (*R*^2^ = 0.98) confirms the quantitative nature of this inhibition.

Control experiments were conducted to rigorously evaluate the specificity of the GLY–CuP interaction. Bare graphite electrodes exhibited no measurable response to GLY exposure, confirming that the observed effects require the CuP modification. The inhibition process demonstrated complete reversibility when electrodes were washed with acidic buffer (pH 3.0) and reactivated in fresh PBS, indicating that GLY binding occurs through specific, non-destructive coordination to the Cu(ii) centers. Furthermore, potential interfering species including nitrate and calcium ions showed minimal impact on sensor response, producing less than 5% signal variation even when present at concentrations ten-fold higher than GLY. These controls collectively verify that the detection system responds selectively to GLY through its unique interaction with the CuP catalytic centers, rather than through non-specific adsorption or interference from common water constituents.

These results demonstrate that the CuP film not only serves as an effective peroxidase mimic but also provides selective recognition sites for GLY detection through competitive inhibition. The combination of catalytic activity and molecular recognition makes this system particularly suitable for environmental monitoring applications where selective herbicide detection is required.

### 3.4 Electrochemical response to GLY

To assess the sensing capability of E/CuP electrodes, we investigated their performance under relevant conditions (pH 7.2 phosphate buffer), which optimally support both the biomimetic peroxidase activity of CuP and GLY coordination chemistry.^21,23^ This pH was selected because glyphosate is fully deprotonated and forms a 1 : 1 complex with Cu^2+^ (Cu-Gly 1 : 1), the most stable species in solution.^19^ Given that the porphyrin structure maintains Cu^2+^ in a redox-accessible state, this ensures efficient coordination with glyphosate. The phosphate buffer was chosen not only for its biocompatible pH (matching peroxidase activity) but also because its ionic strength stabilizes the electrochemistry of the system without disrupting the formation of the Cu(ii)-GLY complex.

**Fig. 4:**
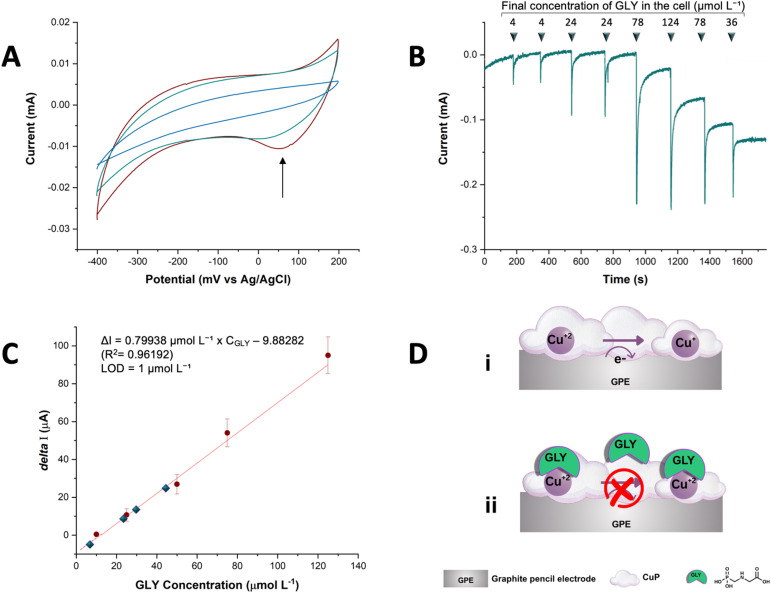
(A) CV of E/CuP recorded in phosphate buffer (pH 7.2, 250 mM) in the presence and absence of GLY. Scan rate: 50 mV s^−11^. Red without GLY, green 5 μmol L^−1^ GLY, blue 10 μmol L^−1^ GLY. (B) Amperometric analysis of the E/CuP electrode in phosphate buffer (pH 7.2, 250 mM) saturated with dissolved O_2_, at an applied potential of +65 mV *vs.* Ag/AgCl. Successive additions of a 1 mM GLY solution resulted in a stepwise decrease in current. (C) The calibration plot shows Δ*I* (*I*_0_ − *I*), where *I*_0_ is the initial current in buffer and *I* is the current after each GLY addition with the real-sample values (from Table S3) overlaid as filled rhombuses. (D) Schematic representation of the sensor's mechanism.

CV revealed a characteristic redox signal at +65 mV *vs.* Ag/AgCl (Fig. 4A), corresponding to the oxygen reduction activity mediated by Cu(ii) centers in the porphyrin matrix. This signal, consistent with literature reports for similar Cu-porphyrin systems,^24,25^ arises from the reversible Cu(ii)/Cu(i) redox transition that drives catalytic oxygen reduction. The progressive attenuation of this peak with increasing GLY concentrations (2–120 μM) directly demonstrates the inhibitory effect of GLY binding, which occupies the catalytic Cu(ii) sites. While GLY is not electroactive in the applied potential range, its coordination to Cu(ii) modifies the redox properties of the metalloporphyrin, altering the electron transfer kinetics at the electrode surface.^35^ The observed amperometric signal reflects the suppression of the electrocatalytic reduction of dissolved oxygen, which is normally mediated by the Cu(ii) site. As GLY binds and inhibits this catalytic activity, a proportional decrease in current is observed, enabling its quantification.

Based on this inhibitory effect, we evaluated the E/CuP electrode as a quantitative sensor using amperometric measurements (Fig. 4B). Successive aliquots of a 1 mM GLY solution were added to 4 mL of 250 mM phosphate buffer (pH 7.2), and the current response was recorded at a fixed potential of +65 mV *vs.* Ag/AgCl. Calibration was performed by plotting the net current response (Δ*I* = *I*_0_ − *I*) *versus* GLY concentration, where *I*_0_ is the baseline current in buffer and *I* is the current after each successive GLY addition. The sensor exhibited a linear response to GLY concentrations ranging from 2 to 120 μmol L^−1^, with a LOD of 1 μmol L^−1^, calculated using the signal-to-noise ratio method (S/N = 3), where the noise was estimated from the standard deviation of the baseline current (Fig. 4C).

Selectivity was assessed by testing the sensor response in the presence of common potential interferents, including nitrate, calcium ions, and humic acid. Selectivity tests showed <5% signal variation for NO_3_^−^, Ca^2+^, and humic acid (10-fold excess), underscoring specificity for GLY.

The microglobular morphology of the CuP film, as observed by SEM (Fig. 1B and C), contributes significantly to the sensor's performance. This nanostructured architecture not only increases the available Cu(ii) binding sites for GLY coordination but also enhances molecular recognition through selective steric exclusion of potential interferents such as nitrate and calcium ions.^36^ The combination of increased binding site density and selective molecular recognition accounts for the sensor's excellent sensitivity and selectivity, as demonstrated in both electrochemical and spectroscopic assays.

Control experiments with a bare electrode in phosphate buffer (Fig. S10) confirmed the absence of redox signals or interference from GLY alone, underscoring that the observed responses arise solely from the CuP-modified electrode. This was further corroborated by amperometric measurements of the bare electrode under identical conditions, which showed no current changes upon GLY addition, confirming the lack of non-specific interactions.

### 3.5. Analytical performance and real-sample validation

Sensor stability was evaluated by storing the modified electrodes at room temperature in the dark and reassessing their response after 30 days. After this period, the sensor retained 99.8% of its initial current signal, demonstrating excellent stability over time.

The sensor's applicability to real matrices was validated using spiked samples prepared with rainwater. Direct amperometric measurements were performed, and the results were compared with nominal concentrations. A matrix-based accuracy of 99.05% was obtained (Table S3), confirming the sensor's applicability to complex aqueous environments.

It is worth noting that the strong binding affinity of GLY to the CuP film may hinder desorption. However, the fabrication cost of each E/CuP electrode is approximately USD 0.12 (see SI), making this platform highly accessible and suitable for field deployment by non-specialized users. While small-scale preparation results in higher per-unit costs due to the absence of industrial efficiencies, mass production could significantly reduce costs, provided there is adequate investment in process automation and standardization.

Limitations can be that irreversible GLY binding necessitates single-use electrodes, though low cost mitigates this. Preliminary electrochemical regeneration was attempted by exposing the used modified electrodes to an acidic solution 0.1 M HCl for 30 min, 1, 2 y 6 hours. While this treatment seemed to restore approximately 65% of the initial current response of the electrode, subsequent measurements showed no sensitivity to GLY. These results suggest that while partial electrochemical reactivation is possible, the structural integrity of the CuP film becomes compromised during regeneration, making the single-use approach more reliable for quantitative applications. Future work could explore regeneration protocols.

While recent studies employ Cu-porphyrin MOFs^5,20^ or nanozymes^23^ for GLY detection, our sensor uniquely integrates electropolymerized CuP films with disposable graphite electrodes, achieving comparable sensitivity (1 μM LOD) without costly nanomaterials or light-dependent signal transduction (Table 2). This design aligns with the need for field-deployable, low-cost herbicide monitoring.

**Table 2:** Comparison of Cu-porphyrin-based GLY sensors

Feature	This work (E/CuP)	Jiang *et al.* (2022)^5^ (CuTCPP/AuNPs/CP)	Zhao *et al.* (2024)^20^ (CuTCPP/C60)	Shen *et al.* (2024)^23^ (CuP-AgNPs)
Platform	Electropolymerized CuP on graphite pencil	Cu-porphyrin MOF + AuNPs on carbon paper	Cu-porphyrin MOF + C60 nanocomposite	Cu-porphyrin nanozyme + AgNPs
Detection method	Amperometry (+65 mV)	Differential pulse voltammetry (DPV)	Photoelectrochemical (PEC)	Colorimetric (absorbance)
LOD (μM)	1	0.15	0.03	0.2
Linear range (μM)	2–120	0.5–100	0.1–50	0.5–100
Selectivity	High (*vs.* NO_3_^−^, Ca^2+^, humic acid)	Moderate (tested *vs.* glucose, urea)	High (*vs.* Pesticides)	Moderate (tested *vs.* ions)
Stability	30 days (99% signal retention)	15 days (85%)	Not specified	7 days (90%)
Fabrication cost	$0.12/electrode	∼$3–5/electrode (AuNPs + MOF)	∼$4–6/electrode (C60 + MOF)	∼$2–3/test (AgNPs)
Real-sample testing	Rainwater (99% recovery)	Lake water (95% recovery)	Not tested	Agricultural runoff (92% recovery)
Key advantage	Low-cost, biomimetic, field-deployable	High sensitivity (DPV)	Ultra-low LOD (PEC)	Rapid visual detection

## 4. Conclusions

In this work, we successfully developed a low-cost, biomimetic electrochemical sensor for GLY detection, employing electropolymerized copper porphyrin (CuP) films deposited on disposable graphite electrodes. The sensor leverages the unique peroxidase–mimetic activity of CuP, enabling indirect GLY quantification through the suppression of Cu(ii)-mediated oxygen reduction upon selective GLY coordination. This innovative approach overcomes the inherent non-electroactivity of GLY, achieving a sensitive detection limit of 1 μM in aqueous samples under neutral pH and ambient conditions. Comprehensive characterization *via* SEM-EDS, Raman spectroscopy, and UV-Vis confirmed the successful deposition of CuP and its specific interaction with GLY, while electrochemical studies (CV, EIS) elucidated the charge-transfer inhibition mechanism underlying the sensor's response.

The sensor demonstrated exceptional analytical performance, including 99% accuracy in spiked rainwater samples, remarkable long-term stability (>99% signal retention over 30 days), and strong selectivity against common interferents such as nitrate and humic acid. With a fabrication cost of just $0.12 per electrode—10–20 times lower than MOF-based alternatives—this platform offers a practical and scalable solution for environmental monitoring. While the irreversible binding of GLY currently necessitates single-use electrodes, their affordability and ease of production make them highly viable for field applications. Future work will focus on integrating the sensor into portable devices, expanding its applicability to other Cu(ii)-binding pollutants, and exploring regeneration strategies to enhance reusability.

By integrating biomimetic design with cost-effective electroanalysis, this study not only advances GLY detection but also establishes a versatile framework for the development of metalloporphyrin-based sensors for environmental and agricultural monitoring.

## Author contributions

Conceptualization: A. M. J. T., R. M. C., M. H. (lead); methodology: A. M. J. T, M. H.; investigation: A. M. J. T., P. G., F.·C.·H., M. H.; formal analysis: A. M. J. T., M. H.; writing – original draft preparation: A. M. J. T., R. M. C., M. H.; writing – review and editing: F.·C.·H., M. H.; supervision: M. H.; funding acquisition: M. H.

## Conflicts of interest

There are no conflicts to declare.

## Supplementary Material

RA-015-D5RA05306D-s001

## Data Availability

Data supporting the findings of this study are available within the article and its SI. See DOI: https://doi.org/10.1039/d5ra05306d.
