# Supplementary material for: Low-cost biomimetic sensor based on copper porphyrin-modified graphite electrodes for electrochemical detection of glyphosate in aqueous samples
Source: RSC Adv. 2025 Aug 26;15(37):30482–9. doi: 10.1039/d5ra05306d (PMC12380045; doi:10.1039/d5ra05306d)
Supplement: RA-015-D5RA05306D-s001 [file RA-015-D5RA05306D-s001.pdf]

Supplementary Information (SI) for RSC Advances.
This journal is © The Royal Society of Chemistry 2025

Supporting Information

Low-cost biomimetic sensor based on copper porphyrin-modified graphite electrodes for electrochemical detection of glyphosate in aqueous samples

Ana M. Janeiro Tudanca;[a] *Rolando M. Caraballo;*[b] *Facundo C. Herrera;*[c] *Paula Giudici*[d, a] *Mariana Hamer*[e]*

[a]Instituto de Ciencias, Universidad Nacional de General Sarmiento, Los Polvorines, Argentina.
[b]INEDES, UNLu-CONICET, Luján, Argentina
[c]Laboratorio Argentino Haces de Neutrones-CNEA, Villa Maipú, Argentina
[d]Instituto de Nanociencia y Nanotecnología, CNEA-CONICET, Centro Atomico Constituyentes, San Martín, Argentina.
[e]Instituto de Ciencias, Universidad Nacional de General Sarmiento-CONICET, Los Polvorines, Argentina

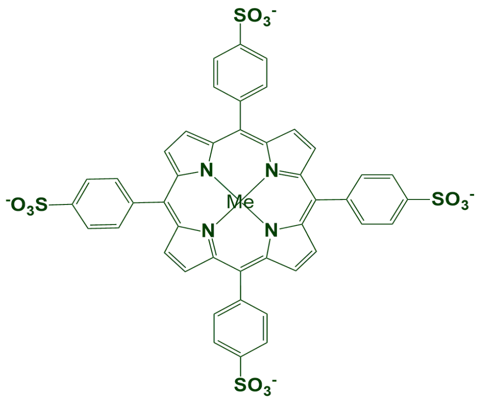

Figure S1. Molecular structure of copper(II) 5,10,15,20-[meso-tetra(4-sulfonatophenyl)porphyrin] (CuP). The central copper ion (Cu) is coordinated to the porphyrin macrocycle.

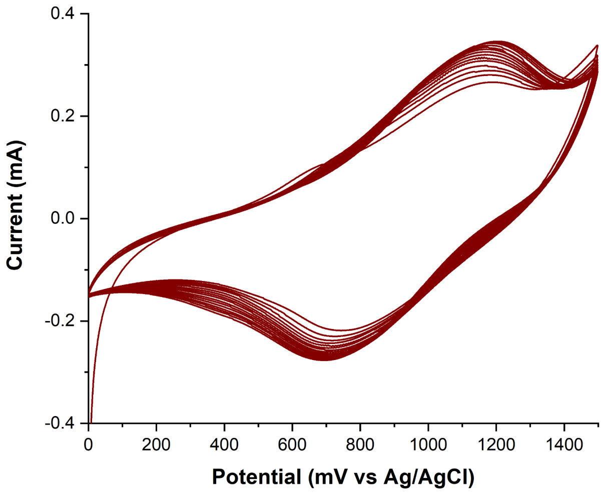

Figure S2. Cyclic voltammetry (CV) scans (0.0 to +1.4 V vs. Ag/AgCl, 50 mV s^{-1}) recorded during the electropolymerization of CuP onto graphite electrodes in 0.1 M NaOH, showing progressive film growth.

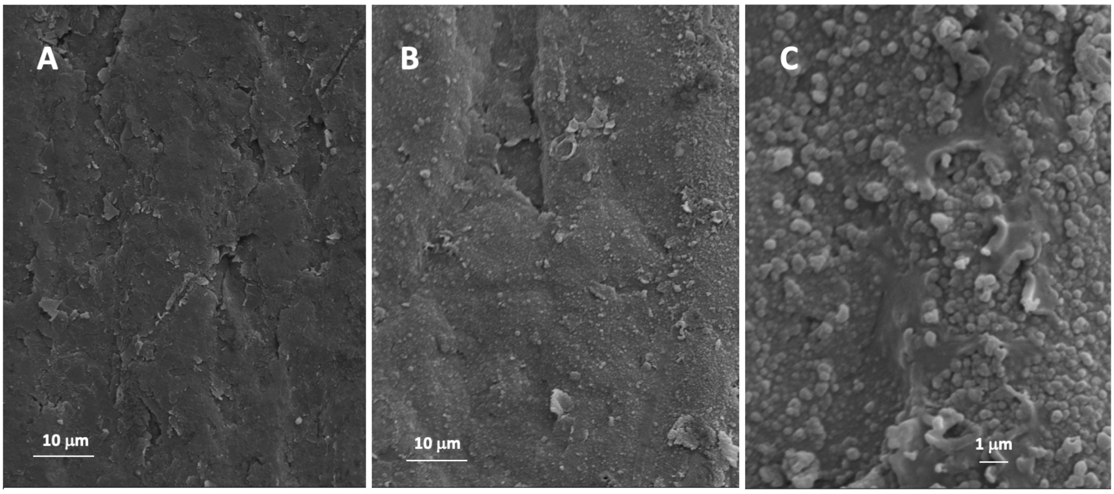

Figure S3. Scanning electron microscopy (SEM) images of (A) bare graphite electrode (E) and (B, C) graphite electrodes modified with electropolymerized CuP (E/CuP). The microglobular morphology of CuP enhances surface area and active sites for GLY detection.

Table S1. Elemental composition (weight % and atomic %) of CuP-modified graphite electrodes (E/CuP) determined by energy-dispersive X-ray spectroscopy (EDS). Key components: Cu (0.8% wt) and S (5.45% wt) confirm successful CuP deposition.

Element	E/CuP	
	**%Weight**	**%Atomic**
C K	79.29	89.16
O K	10.34	8.73
P K	1.30	0.56
Cl K	0.39	0.15
K K	2.42	0.84
Cu K	0.8	0.18
S K	5.45	0.37
Total	100	

NOTE: The presence of K, Cl, and P is attributed to trace ions naturally present in the commercial pencil lead matrix and is not related to the porphyrin deposition process. The successful deposition of the sulfonated copper porphyrin is supported by the clear presence of both copper and sulfur, consistent with the structure of the Cu-porphyrin complex used for the modification.

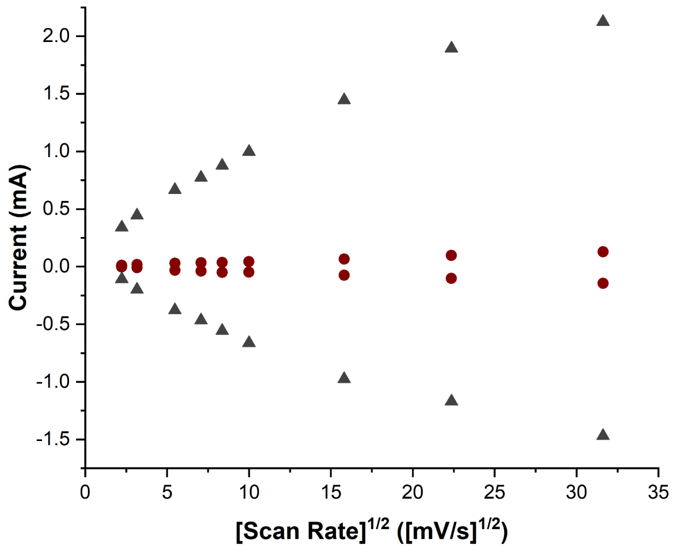

Figure S4. Linear dependence of peak current (Ip) on the square root of scan rate ($v^{1/2}$) for the bare (E, red circles) and CuP-modified (E/CuP, grey triangles) electrodes in 25 mM $Fe(CN)_6^{3-}/Fe(CN)_6^{4-}$ in 0.1 M KNO_3. The linear trend ($R^2 > 0.99$) confirms diffusion-controlled electron transfer, as described by the Randles-Sevcik equation.

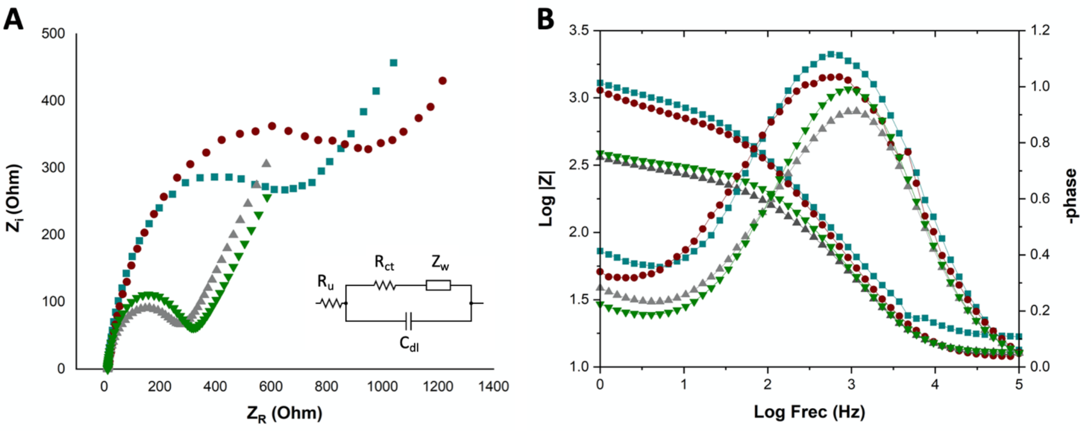

Figure S5. Electrochemical impedance spectroscopy (EIS) analysis: **(A)** Nyquist and **(B)** Bode plots for bare (E, grey triangle), GLY-exposed bare (E+GLY, green triangle), CuP-modified (E/CuP, red circle), and GLY-exposed CuP-modified (E/CuP+GLY, blue square) electrodes in 5 mM $Fe(CN)_6^{3-}$/ $Fe(CN)_6^{4-}$. Inset: Equivalent circuit model used for fitting (R_u = solution resistance, R_{ct} = charge transfer resistance, C_{dl} = double-layer capacitance, Z_w = Warburg impedance).

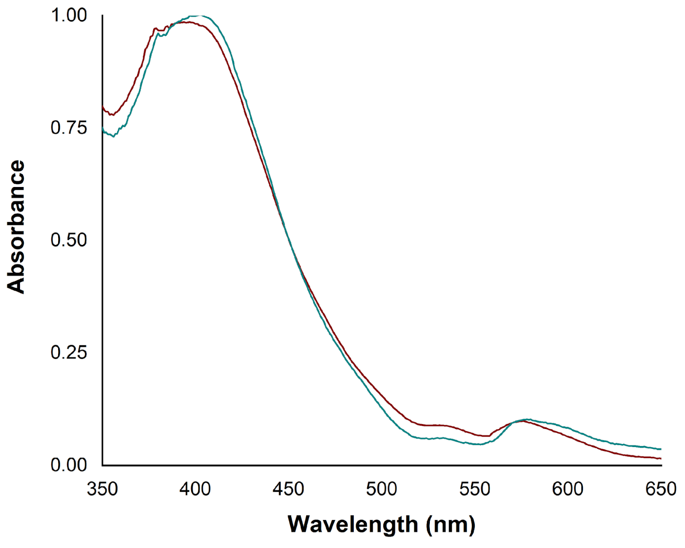

Figure S6. UV-Vis absorption spectra of CuP films on ITO before (red) and after (green) exposure to GLY (100 μmol L^{-1}) in phosphate buffer (pH 7.2). The red shift (~5 nm) in the Soret band (401 → 406 nm) confirms GLY coordination to Cu(II) centers.

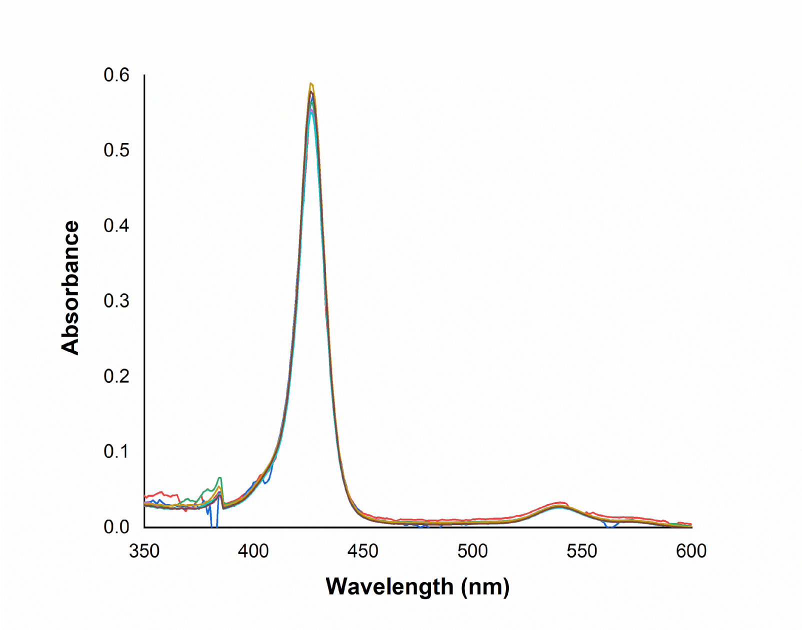

Figure S7. UV-Vis spectra of 20 μmol L^{-1} CuP in solution (PBS, pH 7.2) with increasing GLY concentrations (0–100 μmol L^{-1}). No spectral shifts were observed, suggesting that GLY binding occurs only in the immobilized film form.

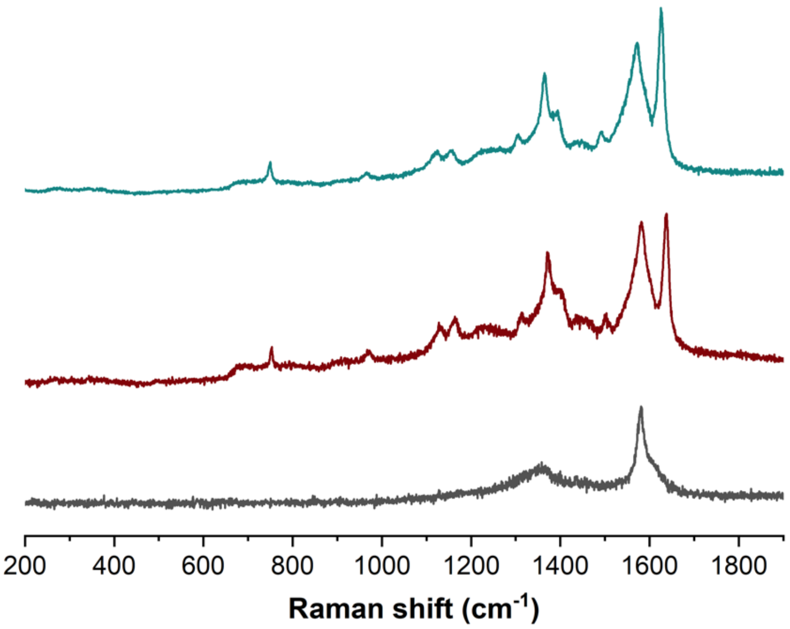

Figure S8. Raman spectra of bare graphite (E, gray), CuP-modified (E/CuP, red), and GLY-exposed CuP-modified (E/CuP+GLY, green) electrodes (λ_{exc} = 514 nm). Key vibrational modes shift upon GLY binding (see Table S2).

Table S2. Raman vibrational mode assignments (cm^{-1}) for CuP films (E/CuP) and GLY-bound CuP (E/CuP+GLY). Downshifts (10-12 cm^{-1}) in C=C and Cα-N stretching modes (e.g., 1638 → 1626 cm^{-1}) indicate porphyrin ring distortion due to Cu(II)-GLY coordination. λexc = 514 nm.

E/CuP	E/CuP + GLY	Assignation
750	750	ν (pyr breathing) non-totally sym
969	968	δ (porph def)
		+ δ (pyr transl)
1028	1025	ν ($C_β$-$C_α$ (vinyl)+ δ($C_β$-R)
1165	1155	δ $C_β$ – H
1312	1304	ν porphyrin / δ ($C_α$H =)
1370	1362	ν $C_α$-N sym
1400	1395	δ (= $C_βH_2$)sym
1501	1490	ν sym C-C + δ asym porphyrin
1581	1573	ν C-C sym
1638	1626	ν ($C_α$ = $C_β$)

ν, stretching; δ, deformation

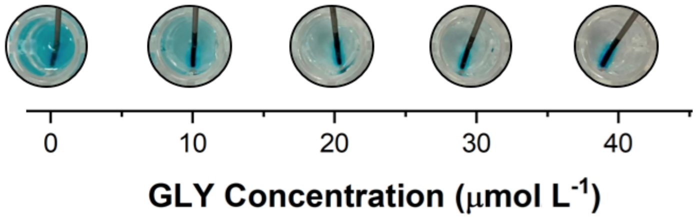

Figure S9. Peroxidase-mimetic activity assay: Peroxidase-mimetic activity assay: Photographs of the reaction mixtures containing oxidized TMB (TMB_{ox}) after 10 minutes of exposure to CuP-modified electrodes, in the presence of increasing concentrations of GLY (0–40 µmol L^{-1}). The progressive fading of the blue color indicates a dose-dependent inhibition of the peroxidase-like catalytic activity by GLY.

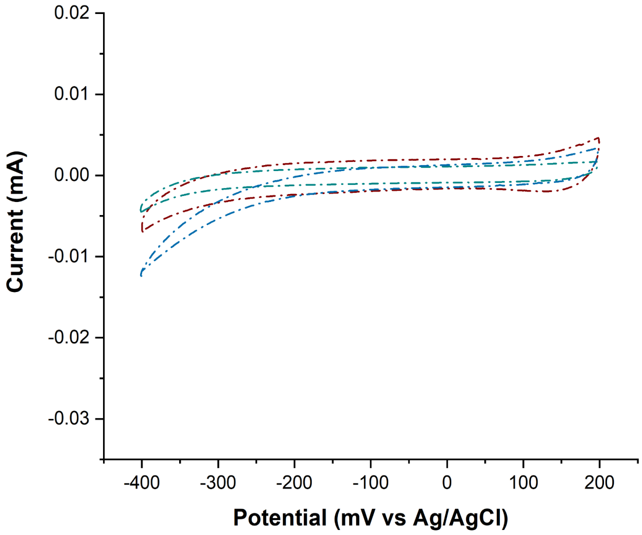

Figure S10. Cyclic voltammograms of bare electrode in phosphate buffer (pH 7.2) without (red) and with GLY (5 µmol L^{-1}: green; 100 µmol L^{-1}: blue).

Table S3. Accuracy and precision of GLY detection in synthetic rainwater samples (n = 3) using E/CuP electrodes. Accuracy rates (98.4–99.8%) and low RSD (<1%) validate sensor reliability in complex matrices.

Sample	Added (µmol L^{-1})	Found (µmol L^{-1})	Accuracy (%)	RSD (%)
**1**	0	0		
**2**	1.50	1.476	98.4	0.9
**3**	45.00	44.910	99.8	0.2
**4**	30.00	29.670	98.9	0.8
**5**	25.00	24.775	99.1	0.7

Note: Accuracy evaluation of the E/CuP sensor in spiked rainwater samples. GLY was added to 4 mL of 250 mM phosphate buffer (pH 7.2) containing 1:1 diluted rainwater. Found concentrations represent the average ± standard deviation (n = 3). Accuracy was calculated as (Found / Added) × 100.

Cost analysis of fabricating a graphite electrode coated with polymerized porphyrin

The base material, a graphite pencil lead, is highly affordable, with an estimated cost of approximately USD 0.10 per unit (e.g., Staedtler graphite slide). The most significant expense arises from the copper porphyrin (CuP), priced at USD 152 per gram (Frontier Scientific), and the alkaline polymerization medium, 0.1 M NaOH, which costs USD 11.50 per liter (Sigma-Aldrich).

For the fabrication of each electrode, only 1 mg of CuP is dissolved in 4 mL of 0.1 M NaOH, a volume sufficient to prepare approximately 10 electrodes. Based on these values, the cost contribution per electrode is USD 0.0152 for CuP and USD 0.0046 for the NaOH solution.

Therefore, the total estimated cost per electrode is approximately USD 0.12, making this sensor a low-cost and scalable platform for environmental applications.

Breakdown of electrode fabrication costs:

- Graphite pencil lead: $0.10/unit
- CuP (1 mg/10 electrodes): $0.0152/electrode
- NaOH solution (4 mL/10 electrodes): $0.0046/electrode
- Total cost: $0.12/electrode.
